# An Efficient and Adaptable Path Planning Algorithm for Automated Fiber Placement Based on Meshing and Multi Guidelines

**DOI:** 10.3390/ma13184209

**Published:** 2020-09-22

**Authors:** Hong Xiao, Wei Han, Wenbin Tang, Yugang Duan

**Affiliations:** 1 State Key Lab for Manufacturing Systems Engineering, Xi’an Jiaotong University, Xi’an 710049, China; jx18hanwei@stu.xjtu.edu.cn (W.H.); tangwb@xpu.edu.cn (W.T.); ygduan@xjtu.edu.cn (Y.D.); 2 School of Mechanical and Electrical Engineering, Xi’an Polytechnic University, Xi’an 710048, China

**Keywords:** fiber-reinforced polymers, automated fiber placement, path planning

## Abstract

Path planning algorithms for automated fiber placement are used to determine the directions of the fiber paths and the start and end positions on the mold surfaces. The quality of the fiber paths determines largely the efficiency and quality of the automated fiber placement process. The presented work investigated an efficient path planning algorithm based on surface meshing. In addition, an update method of the datum direction vector via a guide-line update strategy was proposed to make the path planning algorithm applicable for complex surfaces. Finally, accuracy analysis was performed on the proposed algorithm and it can be adopted as the reference for the triangulation parameter selection for the path planning algorithm.

## 1. Introduction

Fiber-reinforced polymers (FRPs), especially carbon fiber reinforced polymers (CFRPs), are widely used in the aerospace and automobile industry as well as other fields because of their high specific strength, high specific modulus, excellent corrosion- and fatigue-resistance, and outstanding designability [1]. Automated fiber placement combines special manufacturing equipment with computerized numerical control (CNC) systems, which enables good forming quality, strong adaptability, and high efficiency. As an advanced composite manufacturing technology of large and complex components, automated fiber placement has become one of the most advanced and cutting-edge technologies for composite forming [2]. Path planning algorithms for automated fiber placement are used to specify the direction of a fiber path and determine the start and end positions of the fiber on the mold surface. The quality of the fiber path plays a crucial role for the efficiency and quality of the fiber placement process.

**Figure 1:**
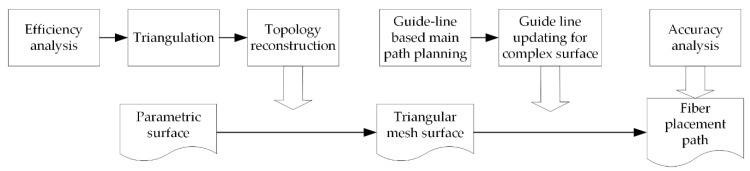
The outline of the proposed algorithm for automated fiber placement.

At present, the typical fiber path planning algorithms include the geodesic method and the meshing method based on parametric surfaces. The path planning algorithm of geodesic method based on parametric surfaces includes analytical and numerical methods [3]. The fiber path, which is obtained via the numerical solution of geodesics, is more aligned with practical engineering requirements. Lewis et al. [4] proposed a path planning method based on natural path, which is also widely used for path planning of tape laying. Shirinzadeh et al. [5,6,7] put forward the method of intersecting lines and surfaces to build the initial path, which improves the adaptability of the path to the surface curvature. However, there are certain disadvantages with the numerical solution of geodesics, e.g., the difficulty of finding the solution and the low efficiency associated with the calculation [8,9]. The meshing method, which uses the polygonal meshes to describe the complex parametric surfaces, can reduce computational complexity. The deviation of the fiber path correlates with the accuracy of the meshing [10,11]. Shinno et al. [12] proposed an iterative geodesic algorithm for a quadrilateral mesh surface to obtain the fiber path. Li et al. [13] proposed a path planning algorithm which used the mesh information contained in the STL file. However, there is a lack of quantitative analysis of the efficiency of the fiber path planning algorithm based on meshed surfaces. Also, no deviation analysis was performed for the generated fiber paths. Meanwhile, for complex components (surfaces), the design of angle reference direction datum is too simple, which leads to the poor applicability of the algorithm, fiber wrinkles, and eventually, affects the quality and mechanical properties of the fabricated FRP components. In this paper, a path planning algorithm for automated fiber placement based on meshing and multi guide-lines was proposed. Both the efficiency and accuracy of the algorithm were analyzed. The outline of the proposed algorithm is shown in Figure 1.

## 2. Efficiency Analysis and Topology Reconstruction 

### 2.1. Efficiency Analysis of the Proposed Algorithm

A traditional path planning algorithm based on the geodesic method of parametric surface generates fiber paths by solving the direction of each point on the surface. It mainly uses the two geometric numerical solution operations, i.e., the intersection between a surface and a plane and the parallel offset of a curve. Essentially, the parallel offset of a curve is realized by the intersection of the surface at the sampling points and the corresponding offset direction planes.

Commercial CAD/CAM softwares generally use NURBS (Non-Uniform Rational B-Splines) for modelling, and the NURBS surface is represented by the following parameter equations:

(1) $$\{\begin{array}{c} x=s_{x}\left(u,v\right)\\ y=s_{y}\left(u,v\right)\\ z=s_{z}\left(u,v\right) \end{array}$$

(2) $$s\left(u,v\right)=\frac{\sum_{i=0}^{C_{u}}\sum_{j=0}^{C_{v}}W_{i,j}P_{i,j}N_{i,p}\left(u\right)N_{j,q}\left(v\right)}{\sum_{i=0}^{C_{u}}\sum_{j=0}^{C_{v}}W_{i,j}N_{i,p}\left(u\right)N_{j,q}\left(v\right)}$$

where $\Omega_{s}$ is the domain of *s*, $P_{i,j}$ is the control point, $W_{i,j}$ is the weight, $N_{i,p}\left(u\right)$ is the basis function of the *p*th-order B-spline in *u* direction, and $N_{j,q}\left(v\right)$ is the basis function of the *q*th-order B-spline in *v* direction.

Let the plane be represented by an implicit surface equation:

(3) h(x,y,z)=0

where $\Omega_{h}$ is the domain of *h*.

Combining Equation (1) with Equation (3) yields the intersection of plane *h* and surface *s* in domain $\Omega =\Omega_{h}\cap^{\;}\Omega_{s}$. The parameters *u* and *v* of the intersection line satisfy the equation for the intersection line [14]:

(4) $$h\left(s_{x}\left(u,v\right),s_{y}\left(u,v\right),s_{z}\left(u,v\right)\right)=l\left(u,v\right)=0$$

Intersection line *l* is a plane curve in the plane of parameter fields for *u* and *v*. If the surface *s* is the *p* × *q*-th order, the intersection line *l* is the *p* × *q*-th order. Because of the low efficiency of high-precision floating-point calculations, the frequent and high-precision solution of the intersection equation *l* consumes a lot of resources. This decreases both efficiency and stability of the numerical algorithms for the intersection between surfaces and planes. In the path planning algorithm based on the parametric surfaces, the intersection between surfaces and planes needs to be solved frequently. This decreases the efficiency of the path calculation, and for complex surfaces, the equation order is too high to be solved, which leads to the failure of path planning.

For triangular mesh surfaces, the surface is approximately a plane within the mesh cell [15]. The intersection between the surface and the plane can be converted into an intersection between planes, and the straight line in the plane is the result of the intersection.

The intersection line equation of a triangular patch $A_{i}\left(x,y,z\right)=0$ and the plane *h* is:

(5) $$l^{\prime }\left(x,y,z\right)=0$$

(6) $$\{\begin{array}{c} x=x_{0}+mt\\ y=y_{0}+nt\\ z=z_{0}+pt \end{array}$$

The intersection line *l*′ is the first order equation about *t*, and the solution complexity decreases significantly. Since the triangulation algorithm will generate discrete grid planes of different sizes with different model complexity, the algorithm will not increase the order and complexity of equation solution during path generation due to the increase of model complexity. Therefore, the fiber path planning algorithm based on triangular mesh surface can obtain stable numerical solution quickly and efficiently.

### 2.2. Triangulation Algorithm for Parametric Surface

In the triangulation algorithm used in this paper, the edge and inner surfaces of the cell surface are approximated by straight line segments. Hence, the surface is discretized into area strips, and it is further divided into plane triangles. The triangulation results for the surface are generally given as strips of triangles. A strip of triangles is a list of points, such that any three consecutive points define a triangle. A parametric surface can be approximated by a series of triangle strips. 

Due to the approximation process of replacing a curved surface with plane surfaces, the discrete error occurs in the triangulation process for parametric surface. This error is mainly reflected in the distance between the meshed plane surfaces and the original parametric surface. Two triangulation parameters are used to constrain the approximation error: (a) D_l2s_: The maximum distance from the straight-line segment of the discrete triangular area to the original parametric surface. (b) L_seg_: The maximum length of the straight-line segment in the discrete triangular area. 

### 2.3. Sub-surface Boundary Splicing and Surface Topology Reconstruction

NURBS curves and surfaces, which are widely used in CAD/CAM software, have exact mathematical expressions, strong expression ability, good quality, and are easy to control. However, for complex surfaces, NURBS surfaces need to be split, spliced and trimmed. A complex surface is usually composed of multiple cellular surfaces. To reconstruct the topology of the whole complex surface, in addition to the mesh reconstruction of the cellular parameter surfaces, boundary splicing between cellular patches should be performed to obtain the global geometric topology. 

#### 2.3.1. Topology Reconstruction Algorithm for the Triangular Mesh of a Cellular Patch

As mentioned above, a cellular surface can be split into feature sampling points. To restore the surface information, it is necessary to obtain the segmentation results and establish the data structure for the triangular meshes by the vertex aggregation algorithm for triangulation (Algorithm 1).
**Algorithm 1: Vertex aggregation algorithm for triangulation**Input: All vertex sets after the triangulation of a cellular parametric surface. Output: Face ID List, Edge ID List and Point ID List for this cellular parametric surface.1: *Loop all strip, fans and triangles:* 2:           According to the right-hand rule, the vertices in the current discrete cell are stored to form the Point ID List of the current cell parameter surface.3: *Loop all points in the Point ID List* 4:           Every three points in the point table form a triangle patch to summarize the Face ID list and store the indexes of the three points of the current triangle patch. 5:           Point ID List update, add triangle patch ID index. 6: Build the edge ID list, update the two-point indexes of the edge, update the Point ID list to add the edge index, update the Face ID list to add the included edge index. 7: Calculate the normal vector of the current triangular patch according to the right-hand rule, and update it in the Face ID list.

Through the above process, the local Point ID list, Edge ID list and Face ID list are established, which reconstruct the topology information for the current NURBS cellular surface.

#### 2.3.2. Algorithm for Subsurface-Boundary Splicing

Because the mesh discretization results for each cellular parameter surface are independent of each other, and two adjacent cellular surfaces share the same edge, there are duplicate vertices for the adjacent cellular surfaces. It is necessary to remove the duplicate vertices. The subsurface-boundary splicing algorithm includes removing duplicate vertices and updating the global index of the vertices in all the cellular surfaces. 

The brute force algorithm, which traverses the whole Point ID List for the duplicate vertices with the same coordinates with a given vertex and hence delete the duplicate ones, is the most straightforward method. Suppose that the surface is made up of k quadrilateral cellular NURBS surfaces with similar area and all the NURBS surfaces are smooth. Following the triangulation, it was assumed that the triangulation results are all given as strips of triangles to obtain a uniform triangular network. If the number of vertices on the boundary is *a* and *b*, the number of vertices on the cellular surface is *a* × *b*, and the number of all vertices is *N* = *k* × *a* × *b*. The time complexity of the brute force algorithm is *O*(*N*^2^). 

**Figure 2:**
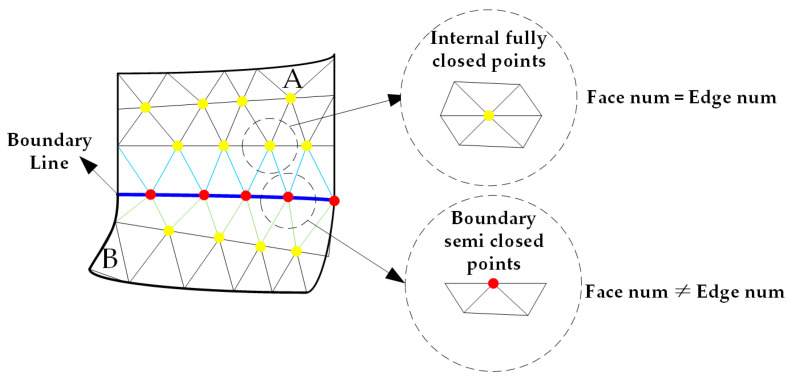
Internal fully-closed points and boundary semi-closed points in cell parametric surfaces.

However, all the duplicate vertices are located on the boundary line because they are formed by two adjacent cellular surfaces sharing a common edge. For the vertices on the boundary line, they are in a semi-closed state and not surrounded by all triangles. On the other hand, the vertices inside the surfaces are in a fully-closed state, surrounded by several triangles (Figure 2). When the vertex is in the fully-closed state, the number of adjacent patches is equal to the number of edges, while in the semi-closed state, the number of patches is not equal to the number of edges. Therefore, all triangle vertices can be divided into two types: boundary semi-closed vertices and internal fully-closed vertices. Thanks to this feature, the boundary vertex set can be filtered out. Then, duplicate vertices can be removed from the boundary vertex set, and Point ID list, Face ID list and Edge ID list can be updated simultaneously.

De-duplication algorithm based on the vertex closure checking was described in Algorithm 2.
**Algorithm 2: De-duplication algorithm based on vertex closure checking**Input: Point ID list before de-duplication. Output: Point ID list after de-duplication. 1: Find semi-closed state vertices 2: *Loop all points in all Point ID List*: 3:           *if point->edgenum != point->facenum* 4:                     save this point to duplicate Point ID List; 5: *Loop all point1 in duplicate Point ID List*: 6:           *Loop all point2 in duplicate Point ID List*: 7:                     *if point1.distanceto (point2) < eps* 8:                               delete point2 in Point ID List; 9:                               refresh Face ID List & Edge ID List;

The time complexity of the algorithm is *O* (*k* × (*a* + *b*)^2^), which is far less than *O* (*N*^2^) of the brute force algorithm, and the efficiency of the algorithm is greatly improved.

At the end of the above process, both sub-surface boundary splicing and surface topological relation reconstruction are completed. This enables fast search of adjacent triangles and efficient path calculation. The topological information of the discrete mesh surfaces is contained in the Point ID list, Face ID list and Edge ID list. The forms and requirements of the three data structure list are shown in Table 1 [13].

## 3. Main Path Planning Algorithm Based on Guidelines on Triangular Mesh Surfaces

The path planning process needs to generate the main motion paths of the head of the automated fiber placement equipment and the corresponding fiber paths which are offset by the main motion paths. The fiber path generation is relatively simple and this paper focuses on the main path planning algorithm. The guidelines are extracted from the CAD model of the mold of the to-be-fabricated FRP component to reflect the skeleton and unique appearance of the component. According to the guide-lines, the direction vector of the main path can be calculated and adjusted adaptively, so that the main path and the corresponding fiber paths can comply with the shape of the component. It is beneficial to improve the mechanical performances of the FRP component while satisfying the constraints of the minimum steering radius of the fiber materials, even distribution of the cutting points, etc. 

### 3.1. Main Motion Path Generation Algorithm

According to the fiber placement process, three geometric parameters (initial starting point *P*_0_, guide line *L*, parametric surface Π) as well as the ply angle *θ* should be provided as input to the main motion path generation algorithm, which outputs the main motion path *l_i_*. Basically, the algorithm needs to calculate the direction vectors and subsequently generate the continuous points on the main motion path, as described in Algorithms 3 and 4, respectively. 

**Algorithm 3: The direction vector calculation algorithm for the main motion path.**

Step 1: Find the projection point *P*_0_’ for the initial starting point *P*_0_ on the triangle patch A of the triangular mesh surfaces ∑ (triangulation of the parametric surface Π) and obtain the normal vector *n* of triangle patch A. Then, redefine *P*_0_’ as the starting point for the current main motion path. 

Step 2: Calculate the tangent vector *t*’ for the projection point *P*_0_’’ of *P*_0_’ on the guide line *L*, and generate the parallel vector *t* of *t*’ at point *P*_0_’.

Step 3: Calculate the orthogonal vector *k* of the normal vector *n* and the parallel tangent vector *t*, where *k* = *n* × *t*. 

**Figure 3:**
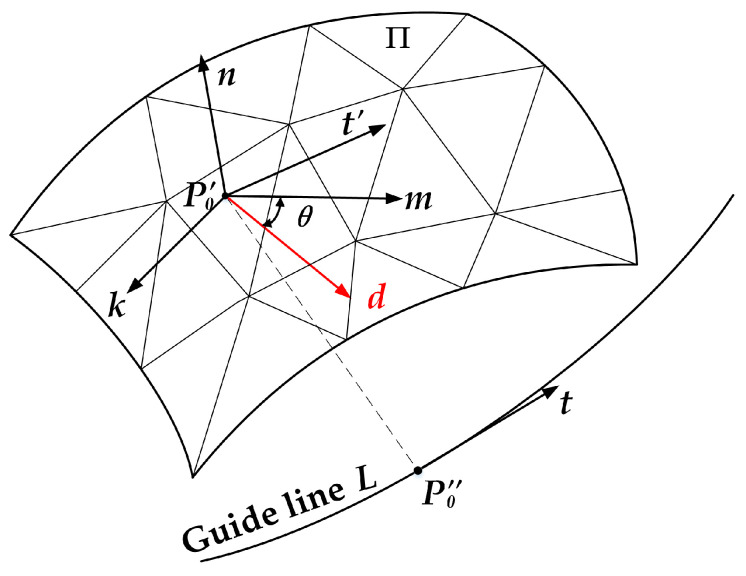
Illustration of the direction vector calculation algorithm.

Step 4: Calculate the orthogonal vector *m* of normal vector *n* and vector *k*, where *m* = *k* × *n*. The vector *m* is the datum direction vector for the current path point, based on the guide-line and corrected by the curvature feature of the surface, where the path point is located (as shown in Figure 3). 

Step 5: Using normal vector *n* as the rotation axis, rotate the datum direction vector *m* for *θ* degree to obtain the laying direction vector *d* for the current path point *P*_0_’.

(7) $$d=\left[\begin{array}{ccc} \cos\theta & -\sin\theta & 0\\ \sin\theta & \cos\theta & 0\\ 0 & 0 & 1 \end{array}\right]\times m$$

In the current triangle patch A, use point *P*_0′_ and vector *d* to construct a straight line and hence obtain the intersection point *P*_1_ of the straight line and the three sides of triangle patch A. The intersection point *P*_1_ is the next path point, and all the path points can be obtained after continuous iteration for the main motion path generation.

**Algorithm 4: The continuous path point generation algorithm.**

Step 1: Take the starting point *P_i_* and direction vector *d_i_* in the current triangle patch *A_i_*, construct a ray *P_a_* (*λ*), which is defined as the solution line for the path point. Its parameter equation is:

(8) $$P_{a}\left(\lambda \right)=P_{i}+\lambda \cdot d$$

Step 2: The vertices *V_a_* and *V_b_* of the triangle patch form a straight line *P_b_* (*λ*), and the parameter equation is:

(9) $$P_{b}\left(\lambda \right)=\left(1-\lambda \right)\cdot V_{a}+\lambda \cdot V_{b}$$

Step 3: Using $P_{a}\left(\lambda \right)=P_{b}\left(\lambda \right)$, we can find the unique solution λ:

(10) $$\lambda =\frac{V_{a}-P_{i}}{d+\frac{\vec{V_{a}-V_{b}}}{\left\|\vec{V_{a}-V_{b}}\right\|}}$$

If 0 ≤ *λ* ≤ 1 is true, this means that the next path point is on the current sideline. If it is not on the current sideline, then take two points to form a straight line for the calculation. Next, take *V_c_V_b_* and *V_a_V_c_* to form a straight line for the calculation. Repeat steps 1, 2, and 3 to get the next path point.

The solution line for the path point may overlap with the three side lines of the triangle in step 3, and the next path point *P_i_*_+1_ cannot be calculated using the above steps. At this time, the endpoint of the edge $V_{m}V_{k}\left(m,k\in \left\{a,b,c\right\}\right)$ of the triangle, where the point *P_i_* is located, is used as the next path point *P_i_*_+1_. The endpoint selection rules are described in Equation (11), where *d* is the laying direction vector.

(11) $$P_{i+1}=\{\begin{array}{c} V_{k},\;d*\bar{V_{m}V_{k}}=1\;\\ V_{m},\;\;\;\;\;\;\mathrm{otherwise} \end{array}$$

Step 4: According to the topological relationship for the triangular mesh surface, obtain the adjacent triangles on the other side of the edge, where *P_i_*_+1_ is located, and update the patch index as *A_i_*_+1_.

Step 5: Update the normal vector *n_i_*_+1_. When the next path point *P_i_*_+1_ lies on the triangle edge or vertex, use the area weighing method to reduce the deviation.

(12) $$n=\frac{\sum_{k=1}^{m}A_{k}\cdot n_{k}}{\left|\sum_{k=1}^{m}A_{k}\cdot n_{k}\right|}$$

In Equation (12), *m* is the total number of adjacent triangles to which *P_i_*_+1_ belongs, and *A_k_* is the area of the triangles. The area can be determined using $A_{k}=\frac{\left\|AB\times AC\right\|}{2}$, where points A, B, C are the three vertices of a triangle *A_k_*. 

Step 6: Update the parallel tangent vector *t_i_*_+1_, and solve the direction vector *d_i_*_+1_ of the next path point *P_i_*_+1_ using Algorithm 3. The projection vector *d_i_*_+1′_ of *d_i_*_+1_ on patch *A_k_*_+1_ is the laying-direction vector of point *P_i_*_+1_.

(13) $$d_{i+1}^{\prime }=d_{i+1}-\frac{d_{i+1}\cdot n}{{\left\|n\right\|}^{2}}\cdot n$$

Step 7: Repeat Step 1 to Step 6 to update and calculate the next path point until reach the triangular mesh surface boundary and no adjacent patch can be found and the partial path on the one single direction is constructed. 

Step 8: Go back to the path initial point *P*_0_, take the inverse of the laying-direction vector *d*_0_ and repeat Step 1 to Step 7 until the whole main motion path is completely constructed.

**Figure 4:**
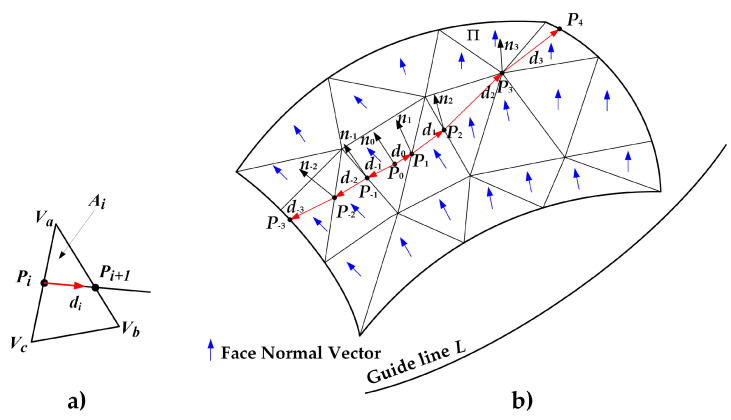
Continuous path point generation algorithm. (**a**) Calculation of next point in single triangle patch. (**b**) Calculation of all points of one path on the triangular mesh surfaces.

The continuous path point generation algorithm is shown in Figure 4. After completing the above steps, the main motion path can be derived using the cubic spline interpolation algorithm.

### 3.2. Guide-line Update Algorithm for Complex Surfaces

**Figure 5:**
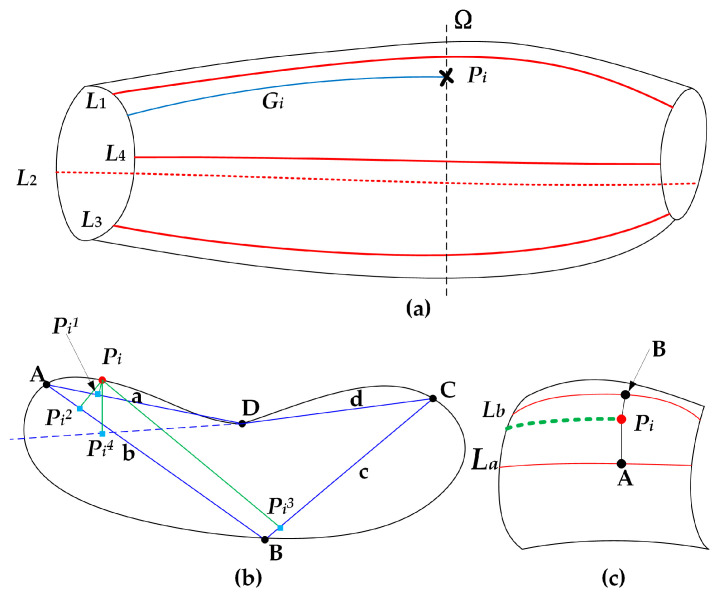
Guide-line update algorithm for complex surfaces: (**a**) Distribution diagram for multi guide-lines. (**b**) Polygonal section formed by the projection point of the point on the guide-line. (**c**) Diagram of tangent vector calculation.

If the curvature of the surface of certain components changes substantially, the path, which was calculated and planned using a single guide line, cannot meet the requirements for the laying ability in each surface area. This leads to wrinkling of the fiber during the laying process, which degrades the mechanical properties of the final fabricated components. To address this problem, this paper proposed a guide-line algorithm for the update of the tangent vector *t* and datum direction vector *m* for the path points according to the surface shape and the distribution of multi guide-lines, as demonstrated in Figure 5 and described in Algorithm 5.

**Algorithm 5: Guideline update algorithm for complex surfaces.**

Step 1: Project the current path point *P_i_* on each guide-line *L_i_* to obtain projection points *A*, *B*, *C* and *D*. Connect the projection points to form a polygonal section plane Ω, the four sides of the polygon on the section plane Ω are *a*, *b*, *c* and *d*.

Step 2: Project the current path point *P_i_* to the edge of the polygon, and find the projection points $P_{i}^{1}$, $P_{i}^{2}$, $P_{i}^{3}$ and $P_{i}^{4}$.

Step 3: If the projection point is on the extension line of the edge, the edge is excluded. As shown in Figure 5b, the projection point $P_{i}^{4}$ is not on the edge *d*, so the edge *d* is not considered in the algorithm below.

Step 4: Calculate the distance from *P_i_* to the projection points on the not excluded edge lines, where $dist=\;\|P_{i}P_{i}^{x}\|$.

Step 5: Determine the minimum distance *dist* and its edge *x*. The two guide-lines *L_a_* and *L_b_*, at the end of *x*, are the guide-lines for the current path point *P_i_*. 

Step 6: As shown in Figure 5c and Equation (14), calculate the tangent vectors on *L_a_* and *L_b_*, respectively. The smooth transition for the path direction vector from *L_a_* to *L_b_* is realized using the distance-weighing method:

(14) $$t=\frac{t_{1}\cdot dist_{1}+t_{2}\cdot dist_{2}}{dist_{1}+dist_{2}}$$

where *dist*_1_ and *dist*_2_ are the distances of *P_i_* to *L_a_* and *L_b_*.

**Figure 6:**
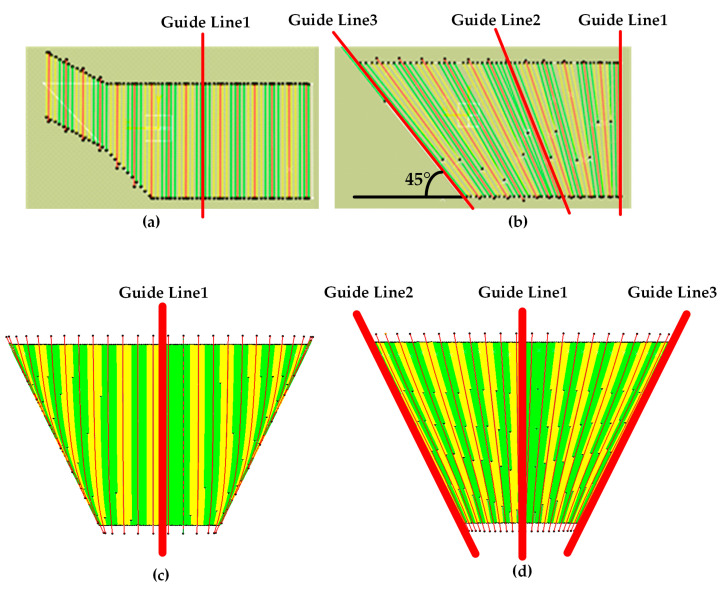
The paths generated based on single guide line and multi-guide lines for a panel (**a**,**b**) and a curved surface (**c**,**d**) models.

Update the tangent vector *t* and calculate the datum direction vector *m* of *P_i_* in Algorithms 3 and 4. The path, which is generated by Algorithm 5, is more adaptive to the changing curvature of the surface and the scalability is improved. Figure 6 shows the paths generated based on single guideline and multi-guidelines for a panel and a curved surface models. It can be seen that the fiber direction transits smoothly between the guide lines and this makes the placed fibers adapt to the shape of the moulds of the final parts. 

## 4. Accuracy Analysis of the Generated Path Based on Surface Meshing

In the triangulation process in Section 2, D_l2s_ and L_seg_ are the main parameters that affect both the mesh density and the approximation accuracy. When the two parameters are set to larger values, the mesh density is small, the surface approximation accuracy is low, the subsequent path planning process data volume is small, and the algorithm efficiency is high. If the two parameter values are continuously reduced, on the other hand, the efficiency is lower.

It is generally believed that the path generated using the geodesic method on the parametric surface is used as the standard path when the planning accuracy and error of the algorithm need to be verified. The points on the discrete surface of the mesh are used to replace the path points on the original parametric surface. Furthermore, the normal vector of the path points on the triangular mesh surface can be used to replace the normal vector on the original parametric surface. As a result, a cumulative error can occur during the iteration process of the proposed path planning algorithm, which can cause the angle deviation from the design datum and the distance deviation from the path generated on the original parametric surface.

**Figure 7:**
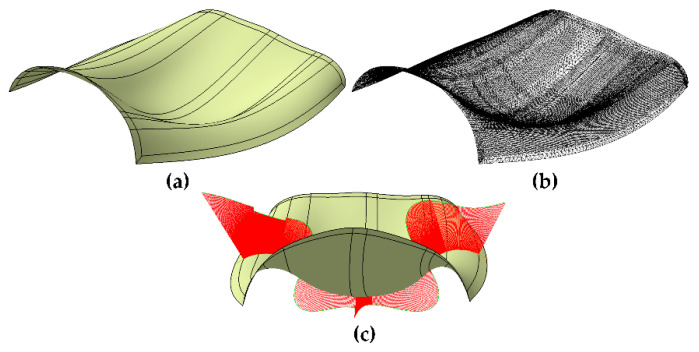
Illustration of the surface for accuracy analysis. (**a**) Original parametric surface. (**b**) Triangular mesh surface with D_l2s_ = 0.2 and L_seg_ = 100. (**c**) Surface curvature variation diagram for the path-deviation-error test area.

A complex surface with positive and negative curvature was adopted to conduct accuracy analysis of the proposed path planning algorithm, as shown in Figure 7. The surface consists of 29 independent cellular patches. Each cellular parameter surface represents a NURBS surface, and the order of each cellular parameter surface in both *U* and *V* directions is 6 degrees. 

### 4.1. Distance Deviation Analysis

**Figure 8:**
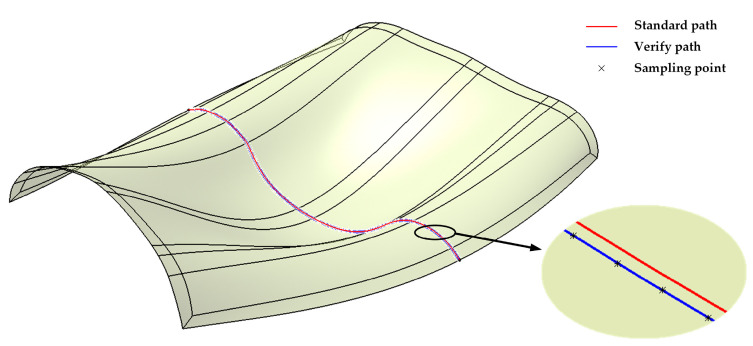
Standard path, verification path generated by the new algorithm, and sample points for D_l2s_ = 0.4 and L_seg_ = 100.

The distance deviation of the path generated on the triangular mesh surface can be decomposed into the normal distance deviation and the geodesic distance deviation. In this paper, a uniform orthogonal test was carried out for the appropriate ranges of D_l2s_ and L_seg_. The path was uniformly sampled (with a distance of 5 mm) to evaluate the distance deviation, as shown in Figure 8.

During the triangulation process, both D_l2s_ and L_seg_ did not reach 0, which means that the parameter value (without approximation error) could not be determined. Therefore, in the actual application, the D_l2s_ range was 0.2–1.0 mm, and the L_seg_ range was 20–200 mm. The experiment was carried out by the $L_{25}\left(5^{6}\right)$ orthogonal design. The path-generation time, the distance deviation, the normal distance deviation and the geodesic distance deviation were recorded.

The Euclidean distance deviation *d* from the sample point on the generated path to the standard reference path was calculated and decomposed into the normal distance deviation *d_N_* and the geodesic distance deviation *d_T_*. 

The project vector from the sample point to standard reference path is *V*, and the normal vector for sample point *P_i_* on the original parametric surface is *n*. *d_N_* and *d_T_* can be calculated as follows:

(15) $$\{\begin{array}{c} d=DistBetween\left(P_{i},P_{i}^{\prime }\right)\\ d_{N}=d\times \cos<V,n>\\ d_{T}=\sqrt{d^{2}-d_{N}^{2}} \end{array}$$

According to the $L_{25}\left(5^{6}\right)$ orthogonal design, 25 sets of experiments were carried out. Four of them, which were D_l2s_ = 0.2 and L_seg_ = 65, D_l2s_ = 0.4 and L_seg_ = 110, D_l2s_ = 0.6 and L_seg_ = 155 and D_l2s_ = 0.8 and L_seg_ = 200, were selected to analyze the distribution of distance deviation along the generated paths.

**Figure 9:**
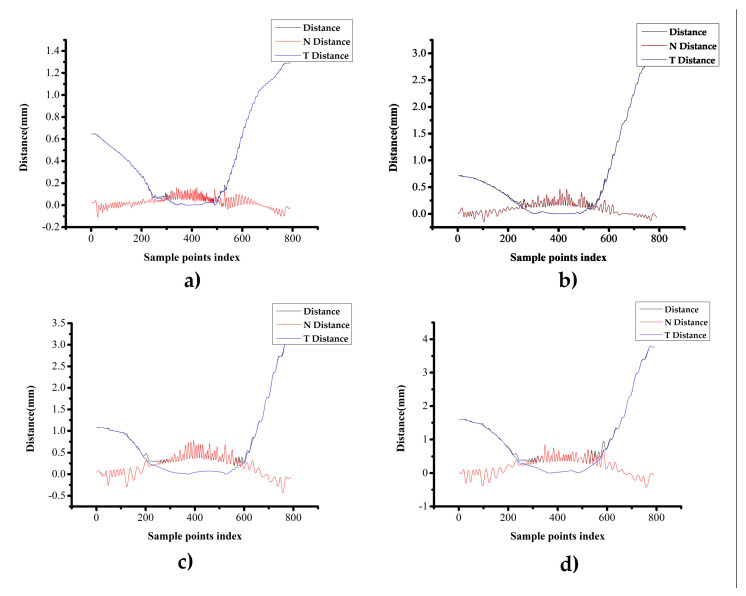
The distance deviation distribution (N Distance is *d_N_* and T Distance is *d_T_*.) for different parameters (**a**) D_l2s_ = 0.2, L_seg_ = 65, (**b**) D_l2s_ = 0.4, L_seg_ = 110, (**c**) D_l2s_ = 0.6, L_seg_ = 155 and (**d**) D_l2s_ = 0.8, L_seg_ = 200.

The initial path point was located near the 350th sample point. According to Figure 9, the closer the sample point was to the initial point, the smaller was the distance deviation. During path generation, the normal vector for the path points on the triangular mesh surface was used to (approximately) replace the normal vector of the path points on the original parameter surface. Therefore, the direction datum of the path point was deviated, and it caused geodesic distance deviation between the generated path and the standard reference path. It also shows that the geodesic distance deviation *d_T_* was the main deviation and it increased as the parameters D_l2s_ and L_seg_ increased. 

To analyze the relationship between the parameters (i.e., D_l2s_ and L_seg_) and the distance deviation of the generated path and the algorithm efficiency, the mean distance deviation, the normal mean distance deviation, the geodesic mean distance deviation, the maximum distance deviation, the maximum normal distance deviation, the maximum geodesic distance deviation, and the path generation time were calculated. This was done when the generation time started from the beginning of the surface triangulation to the end of the path generation, as shown in Table 2.

**Figure 10:**
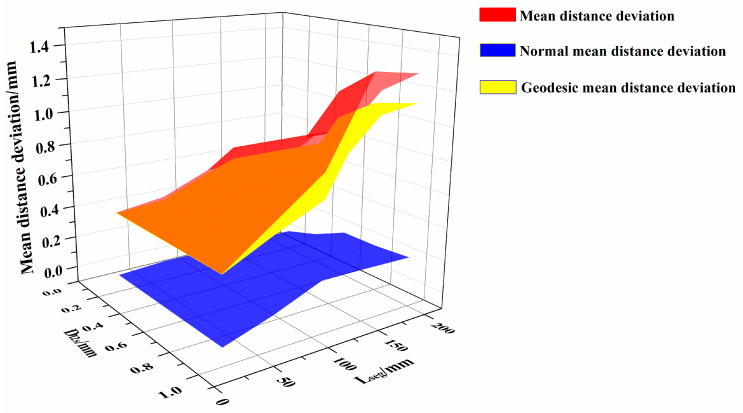
Mean distance deviation and its decomposition.

**Figure 11:**
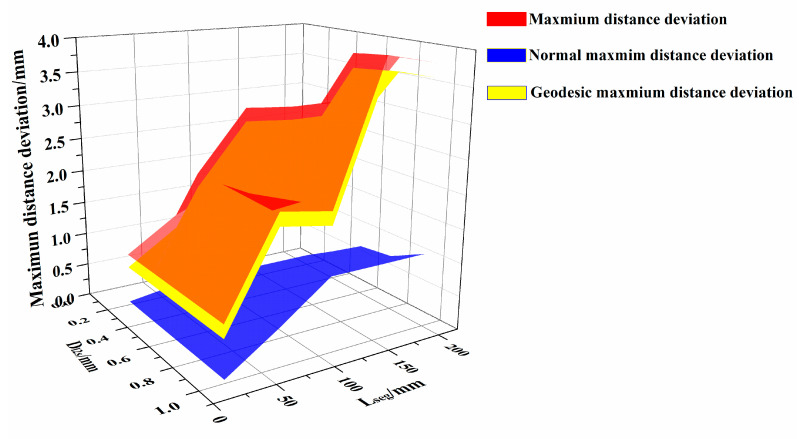
Maximum distance deviation and its decomposition.

**Figure 12:**
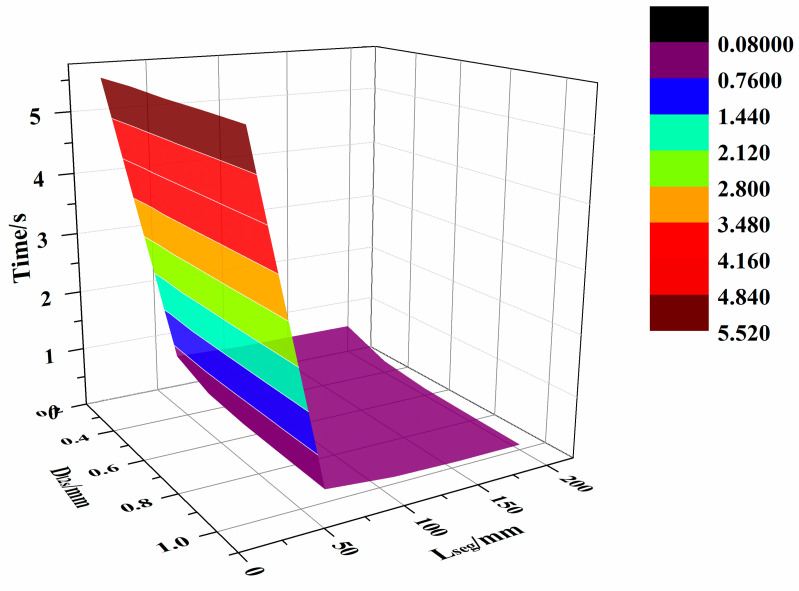
The generation times.

The distribution of mean distance deviation are shown in Figure 10, while the maximum distance deviation are shown in Figure 11, and the generation time are shown in Figure 12. Supplementary Video S1 further demonstrates the high efficiency of the proposed algorithm, which can complete the path planning for one layer of a complex surface in just only tens of seconds.

According to Figure 10 and Figure 11, for different parameters, the distance deviation mainly consisted of geodesic distance deviation, and the effect of normal distance deviation on the overall distance deviation is relatively small. Meanwhile, it can be observed that D_l2s_ and L_seg_ restricted each other in the triangulation process. When the two parameters cannot be satisfied simultaneously, the algorithm will adopt the parameter that makes the mesh more precise. For example, when L_seg_ is 20 mm, the triangulation algorithm generated the same triangular mesh surfaces for a D_l2s_ of 0.4 mm, 0.6 mm, 0.8 mm and 1.0 mm as for the D_l2s_ of 0.2 mm. 

Table 2 shows that, when D_l2s_ exceeds 0.8mm and L_seg_ exceeds 155 mm, the mean distance deviation surpasses 1mm, and the maximum distance deviation exceeds 2 mm. When D_l2s_ is between 0.2 and 0.6mm, and L_seg_ is 65 to 155 mm, the average distance deviation remains within 1mm, while the maximum distance deviation is within 2 mm. The generation time of the algorithm is within 0.5 s.

### 4.2. Angle Deviation Analysis

To ensure the orthogonal arrangement of fibers in different layers, the angle distribution between different layers is strictly defined. This ensures quasi-isotropic mechanical behavior for the components [3]. To analyze the angle deviation between the generated paths and the design datum, an angle deviation analysis was performed in this section.

The generated path was sampled uniformly with a step of 5 mm. For each sample point, the forward direction of the current path and the design direction datum of the path were calculated using both the guide-line and surface normal vector at the point. Subsequently, the angle deviation was obtained. The forward direction vector $d_{i}$ at each sample point is the tangent vector of the sample point on the path. The datum direction vector $d_{i}^{\prime }$ can be calculated using:

(16) $$\{\begin{array}{c} k_{i}^{\prime }=n_{i}^{\prime }\times t_{i}^{\prime }\\ d_{i}^{\prime }=n_{i}^{\prime }\times k_{i}^{\prime } \end{array}$$

where $n_{i}^{\prime }$ is the normal vector of the sample point on the surface, and $t_{i}^{\prime }$ is the tangent of the projection point for the sample point on the guide-line. The angle deviation $\delta_{i}$ is calculated using:

(17) $$\delta_{i}=arcos<d_{i},d_{i}^{\prime }>$$

The four sets of experiments selected in Section 4.1 were also adopted here to analyze the distribution of the angle deviation. 

**Figure 13:**
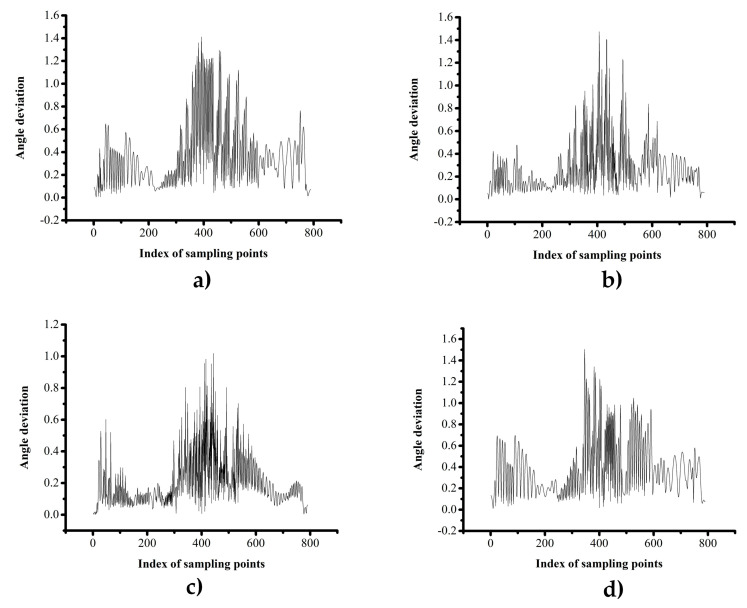
The angle deviation with different parameters: (**a**) D_l2s_ = 0.2, L_seg_ = 65; (**b**) D_l2s_ = 0.4, L_seg_ = 110; (**c**) D_l2s_ = 0.6, L_seg_ = 155 and (**d**) D_l2s_ = 0.8, L_seg_ = 200.

As mentioned above, the initial path point was near the 350th sampling point. According to Figure 13, for different triangulation parameters, the angle deviation does not depend on the distance between the sampling point and the initial path point but depend on the curvature of the original surface. The larger the curvature of the surface, where the sample points are located on the path, the greater is the angle deviation. This confirms that the angle deviation is due to the approximation of the normal vector of the triangular mesh surface during the execution of the algorithm, and there is no cumulative error.

To study the effect of D_l2s_ and L_seg_ on the angle deviation of the path, the mean angle deviation, the mean square error, and the maximum angle deviation were calculated using the experimentally obtained data—see Table 3.

**Figure 14:**
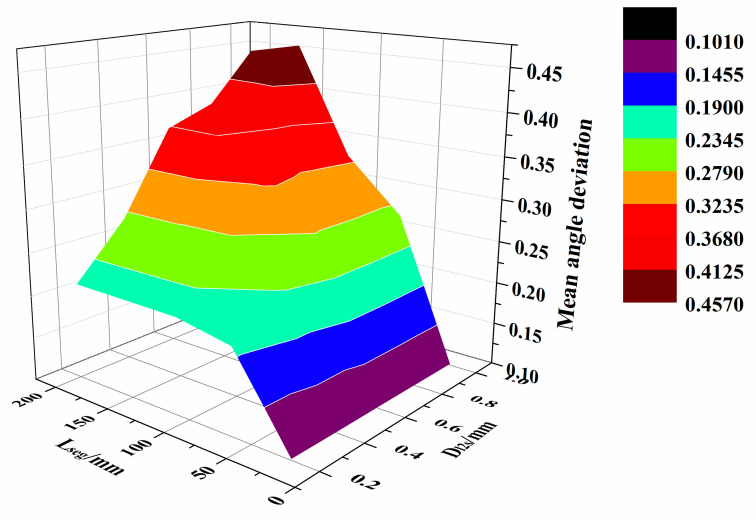
Distribution of the average angle deviation.

**Figure 15:**
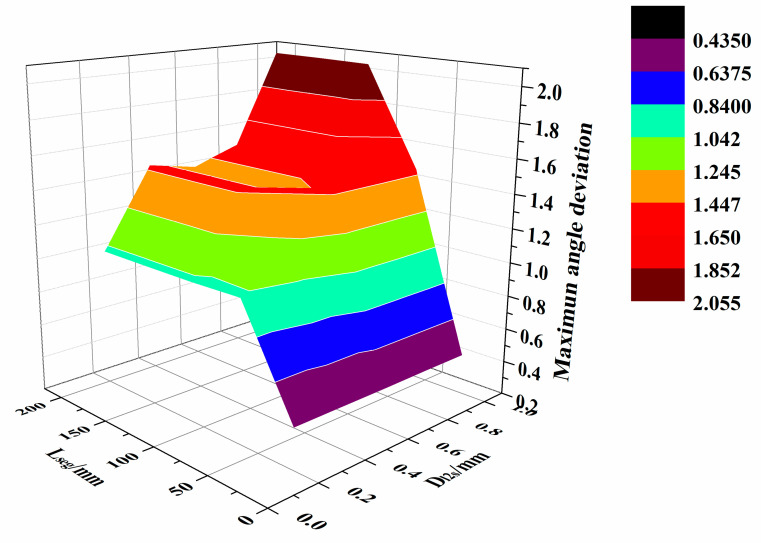
Distribution of the maximum angle deviation.

The distribution of average angle deviation data is shown in Figure 14, and the maximum angle deviation data distribution is shown in Figure 15.

According to Figure 14 and Figure 15, as the D_l2s_ and L_seg_ increased, both the mesh density and approximation accuracy of the triangular mesh surface decrease. In addition, the normal vector of the path points on the triangular mesh surface deviate significantly from the normal vector on the original parametric surface. Hence, the angle deviation increases. According to Table 3, when D_l2s_ exceeds 0.6 mm and L_seg_ exceeds 65 mm, the mean angle deviation surpasses 0.25 deg, and the maximum deviation is more than 1.4 deg.

Based on the above distance deviation analysis, when D_l2s_ ranges between 0.2 and 0.6 mm and L_seg_ is 65 to 110 mm, the mean distance deviation remains within 1mm. Furthermore, the maximum distance deviation stays within 2 mm, the mean angle deviation is less than 0.25 deg, and the maximum angle deviation is below 1.4 deg. At the same time, the path generation time stays within 0.5s. In this case, D_l2s_ and L_seg_ can be selected according to the complexity of the surface and the acceptable path error. Within the above range of the parameters, a high-precision triangular mesh surface and fiber path with small error can be obtained, while the generation efficiency of the algorithm is high.

## 5. Conclusions

To improve the efficiency of automated fiber path planning process, a new path planning algorithm based on meshing and multi guide-lines were investigated. The original parameter surface of the CAD model of the FRP component was discretized into triangular mesh surface via surface discretization and triangulation. Sub-surface boundary splicing and surface topology reconstruction algorithm was proposed, and both the computational complexity reduction and the efficiency improvement of the algorithm were analyzed. The proposed automated fiber path planning algorithm consists of a main motion path direction vector algorithm and a continuous path point generation algorithm. An updating method for the datum direction vector via the guide-lines update algorithm was also introduced for complex surfaces. It improves the laying ability of the fibers and surface adaptability for the planned path. Accuracy analysis was conducted to investigate the relationship between the triangulation parameters and distance deviation, angle deviation and algorithm efficiency. The analysis indicated that by choosing appropriate triangulation parameters, the fiber path can be generated with high accuracy and efficiency. 

More research efforts in the future work should be devoted to conduct experiments to test the mechanical properties of the fabricated FRP components by using the multi-guide-line planned paths. 

## Figures and Tables

**Table 1:** The forms and requirements of the three data structure lists.

Class	Contents	Features
Vertex	int vIndex; double p [3]; int EdgeIndexList [cur edge index]; int FaceIndexList [cur face index];	Given the index number of a vertex, one can quickly find the global index of the triangle patch and the global index for the edge, where the current vertex belongs.
Edge	int eIndex; int VertexIndexList [2]; int FaceIndexList [2];	Given the number of a side, one can quickly find the global index of the face, where the current edge belongs and the global indices of the two vertices at the current edge.
Face	int fIndex; doubla n [3];int VertexIndexList [3]; int EdgeIndexList [3];	Given the number of a triangle patch, one can quickly find the global index and the global index of the three edges of the three vertices on the triangle patch.

**Table 2:** Distance deviation and generation time.

D_l2s_ /mm	L_seg_ /mm	Mean Distance Deviation /mm	Normal Mean Distance Deviation /mm	Geodesic Mean Distance Deviation /mm	Maximum Distance Deviation /mm	Maximum Normal Distance Deviation /mm	Maximum Geodesic Distance Deviation /mm	Generation Time /s
0.2	20	0.41072	0.00824	0.40834	0.81222	0.05218	0.81219	5.506
0.2	65	0.45268	0.02694	0.4329	1.29759	0.16468	1.29745	0.549
0.2	110	0.57411	0.02534	0.55406	1.60354	0.16447	1.60342	0.508
0.2	155	0.57298	0.02557	0.55294	1.60354	0.16447	1.60342	0.508
0.2	200	0.57309	0.02547	0.55304	1.60354	0.16447	1.60342	0.505
0.4	20	0.41072	0.00824	0.40834	0.81222	0.05218	0.81219	5.514
0.4	65	0.54446	0.09072	0.47922	2.12237	0.36747	2.12172	0.296
0.4	110	0.78375	0.09412	0.71355	3.00629	0.46252	3.00561	0.214
0.4	155	0.78403	0.09052	0.71476	2.94274	0.46246	2.94204	0.209
0.4	200	0.78403	0.09052	0.71476	2.94274	0.46246	2.94204	0.213
0.6	20	0.41072	0.00824	0.40834	0.81222	0.05218	0.81219	5.495
0.6	65	0.62143	0.1036	0.55255	2.14027	0.55588	2.13962	0.231
0.6	110	0.65292	0.20369	0.4915	1.62566	0.77333	1.62391	0.142
0.6	155	0.86012	0.18893	0.69804	3.09987	0.77349	3.0993	0.13
0.6	200	0.8602	0.18895	0.6985	3.08182	0.77349	3.08124	0.128
0.8	20	0.41072	0.00824	0.40834	0.81222	0.05218	0.81219	5.499
0.8	65	0.62315	0.10188	0.55424	2.19272	0.55588	2.19207	0.213
0.8	110	0.78546	0.20771	0.63916	1.94612	0.84012	1.94459	0.115
0.8	155	1.18547	0.19293	1.03511	3.91933	0.84004	3.91922	0.098
0.8	200	1.15638	0.19065	1.00657	3.8031	0.84004	3.80299	0.103
1	20	0.41072	0.00824	0.40834	0.81222	0.05218	0.81219	5.491
1	65	0.62147	0.10314	0.55262	2.14027	0.55588	2.13962	0.203
1	110	0.83477	0.2216	0.68092	2.01082	1.10312	2.00955	0.105
1	155	1.33796	0.21533	1.16792	3.97448	1.10346	3.97407	0.082
1	200	1.29659	0.20791	1.12927	3.81894	1.10346	3.8185	0.083

**Table 3:** Data used for the calculation of the angle deviation.

D_l2s_ /mm	L_seg_ /mm	Mean Angle Deviation /deg	Mean Square Error /deg^2^	Maximum Angle Deviation /deg
0.2	20	0.10162	0.09583	0.43632
0.2	65	0.19909	0.1562	1.019
0.2	110	0.21098	0.15039	1.01133
0.2	155	0.21159	0.15056	1.01142
0.2	200	0.21204	0.15059	1.01141
0.4	20	0.10162	0.09583	0.43632
0.4	65	0.22812	0.16134	1.1537
0.4	110	0.27022	0.21115	1.47317
0.4	155	0.27248	0.20807	1.47248
0.4	200	0.27248	0.20807	1.47248
0.6	20	0.10162	0.09583	0.43632
0.6	65	0.2696	0.24432	1.47683
0.6	110	0.33184	0.27956	1.41166
0.6	155	0.36801	0.27632	1.4119
0.6	200	0.36668	0.27738	1.4119
0.8	20	0.10162	0.09583	0.43632
0.8	65	0.27083	0.2446	1.47683
0.8	110	0.3184	0.25209	1.50431
0.8	155	0.39048	0.25162	1.50424
0.8	200	0.38589	0.2524	1.50424
1	20	0.10162	0.09583	0.43632
1	65	0.26882	0.24516	1.47683
1	110	0.33021	0.29158	2.0499
1	155	0.45625	0.32954	2.05242
1	200	0.44346	0.32784	2.05242

## Supplementary Materials

The following is available online at https://www.mdpi.com/1996-1944/13/18/4209/s1, Supplementary Video S1 demonstrates the high efficiency of the proposed algorithm, which can complete the path planning for one layer of a complex surface in just only tens of seconds.
